# Focal lamina cribrosa defects are not associated with steep lamina cribrosa curvature but with choroidal microvascular dropout

**DOI:** 10.1038/s41598-020-63681-6

**Published:** 2020-04-21

**Authors:** Seung Hyen Lee, Tae-Woo Kim, Eun Ji Lee, Michaël J. A. Girard, Jean Martial Mari

**Affiliations:** 1 Department of Ophthalmology, Bundang Jesaeng General Hospital, Daejin Medical Center, Seongnam, Korea; 2 Department of Ophthalmology, Seoul National University College of Medicine, Seoul National University Bundang Hospital, Seongnam, Korea; 3 Department of Biomedical Engineering, National University of Singapore, Singapore, Singapore; 4 Singapore Eye Research Institute, Singapore National Eye Centre, Singapore, Singapore; 5 Université de la Polynésie française, Tahiti, French Polynesia

**Keywords:** Optic nerve diseases, Risk factors

## Abstract

Focal lamina cribrosa (LC) defects have been found to play an important role in the development and progression of glaucomatous optic neuropathy. However, the mechanism of generation of focal LC defects is largely unknown. This cross-sectional study was performed to investigate LC curvature and the frequency of parapapillary choroidal microvascular dropout (MvD) in glaucomatous eyes with focal LC defects. This study was conducted by a retrospective review of patients with primary open-angle glaucoma (POAG) included in an ongoing prospective study being performed at the Seoul National University Bundang Hospital (Investigating Glaucoma Progression Study). A total of 118 eyes of 118 patients with POAG, 59 with and 59 without focal LC defects, with eyes matched by age, axial length, and severity of visual field (VF) damage were included. Posterior LC bowing was assessed by calculating LC curvature index (LCCI), as the inflection of a curve representing a section of the LC, on the optic nerve head images obtained by enhanced-depth-imaging (EDI) spectral-domain optical coherence tomography (OCT). MvD was detected by OCT angiography. LCCI and MvD frequency were compared between eyes with and without focal LC defects. Mean LCCI was significantly smaller than in eyes with than without focal LC defects (9.75 ± 1.29 vs. 11.25 ± 1.39, *P* < 0.001). MvD was significantly more frequent in eyes with than without focal LC defects (84.7% vs. 49.2%, *P* < 0.001). MvD in eyes with focal LC defects showed a strong topographic correlation with the focal LC defects. These findings suggest that focal LC defects may primarily result from vascular factors rather than from mechanical strain.

## Introduction

The lamina cribrosa (LC) is considered to be the primary site of glaucomatous axonal damage^1,2^. Posterior deformation of the LC (i.e., bowing and compression), which has been demonstrated in histologic^3^ and experimental studies^4,5^ may impose shearing stress on the optic nerve axons or hamper axonal transport. In addition, LC compression may induce collapse of capillaries inside the laminar beams leading to optic nerve ischemia. These changes may ultimately promote axonal damage.

Recent improvements in imaging modalities such as enhanced depth imaging (EDI) of spectral-domain optical coherence tomography (SD-OCT) or swept source OCT, have enabled *in vivo* imaging of LC. Of the findings identified using EDI-OCT, focal LC defect has received high levels of attention. Studies reported that focal LC defects were exclusively presented in glaucoma^6^, and were associated with local glaucomatous optic disc appearances such as neuroretinal rim thinning^7^, acquired pits of the optic nerve (APON)^7^, and localized retinal nerve fiber layer (RNFL) loss^8^. In addition, the rate of visual field worsening was faster in eyes with than without focal LC defects^9^. These findings suggested that focal LC defect is likely have important pathogenic relevance to glaucomatous optic neuropathy. Understanding the etiopathology of focal LC defect development may provide insight into the pathogenesis of glaucoma.

OCT angiography has been utilized to assess microvasculature dropout (MvD) in eyes with glaucoma^10^. The prevalence of focal LC defects was found to be higher in eyes with than without MvD, and MvD was found to be topographically associated with focal LC defects^10^. MvD is regarded as a true impairment of perfusion, based on its correspondence to the perfusion defect identified by indocyanine green angiography^11^. These observations suggested that the development of focal LC defect is associated with vascular compromise. However, it is unclear whether stress induced by intraocular pressure (IOP) also plays a significant or more important role than vascular factors in the development of focal LC defects.

Experimental studies have demonstrated that the LC bows posteriorly when the IOP is increased^4,5^. Conversely, the LC became less curved when IOP is lowered in glaucoma patients^12^. In addition, the LC is only slightly curved in healthy eyes compared to glaucomatous eyes, rendering LC curvature to have a high capability to discriminate between glaucomatous and healthy eyes^13^. Taken together, it may be proposed that steep LC curvature can be used as a surrogate representing posterior LC deformation or remodeling induced by translaminar differences of pressure.

We hypothesized that LC morphology would be steeply curved if IOP-induced stress/strain plays an important role in the development of focal LC defect and relatively flat if not. The purposes of the present study were to evaluate LC morphology in glaucomatous eyes with focal LC defects, and to determine the association of focal LC defect with parapapillary choroidal MvD.

## Results

This cross-sectional study included 211 eyes of 203 patients initially. Of these, 51 eyes of 47 patients were excluded due to myopic tilted disc eye with gamma zone, and 21 eyes of 19 patients were excluded because of poor quality SD OCT ONH images, and eight eyes of seven patients were excluded due to poor quality OCTA images. After matching for age, axial length, and mean deviation of visual field test between patients with and without focal LC defects, 59 eyes of 59 patients with POAG were finally included in each group.

Table 1 describes the characteristics of the subjects. There were no significant disparities between patients with and without focal LC defects in baseline clinical, systemic and ocular characteristics, except for frequency of MvD and LCCI at all locations. MvD was significantly more frequent (*P* < 0.001) and LCCIs at all locations were significantly lower (all *P* values ≤ 0.003) in eyes with than without focal LC defects (Table 1)

**Table 1:** Baseline characteristics of the participants.

Variables	Focal LC defect (n = 59)	Control glaucoma (n = 59)	*P* value
**Demographic characteristics**			
Age, years	57.8 ± 10.4	57.7 ± 10.6	0.979
Female (%)	36 (61.0)	29 (49.2)	0.267
**Systemic characteristics**			
Diabetes mellitus, no. (%)	5 (8.5)	5 (8.5)	1.000
Systemic hypertension, no. (%)	15 (25.4)	21 (35.6)	0.318
SBP, mmHg	121.6 ± 9.6	125.9 ± 14.7	0.061
DBP, mmHg	75.0 ± 7.6	76.6 ± 9.7	0.318
MAP, mmHg*	90.5 ± 7.8	93.0 ± 9.9	0.128
MPP, mmHg†	47.2 ± 5.2	48.1 ± 7.2	0.410
**Ophthalmic characteristics**			
Baseline IOP, mmHg	16.7 ± 2.6	17.3 ± 3.1	0.264
ScanIOP, mmHg	13.2 ± 2.0	13.9 ± 2.3	0.071
Spherical error, diopters	−0.76 ± 2.26	−0.89 ± 2.59	0.774
Axial length, mm	23.89 ± 1.10	23.86 ± 1.18	0.888
Central corneal thickness, μm	555.2 ± 29.1	557.5 ± 35.3	0.706
VF MD, dB	−6.84 ± 6.51	−6.95 ± 6.18	0.922
Global RNFL thickness, μm	74.8 ± 14.2	74.2 ± 13.2	0.805
**Presence of MvD (%)**	51 (86.4)	31 (52.5)	**<0.001**
Area of MvD, mm^2^	0.25 ± 0.24	0.13 ± 0.19	**0.002**

LC = lamina cribrosa; SBP = systolic blood pressure; DBP = diastolic blood pressure; MAP = mean arterial pressure; MPP = mean perfusion pressure; IOP = intraocular pressure; VF = visual field; MD = mean deviation; dB = decibel; RNFL = retinal nerve fiber layer; MvD = microvasculature dropout.

Data are reported as mean ± standard deviation, with statistically significant *P* values in boldface.

^*^Mean arterial pressure = diastolic BP + 1/3 (systolic BP - diastolic BP).

^†^Ocular perfusion pressure = 2/3 (mean arterial pressure) − scan IOP.

The 95% Bland-Altman limits for interobserver agreement of measuring the 826 LCCIs (i.e., seven B-scans of 118 eyes) by the two glaucoma specialists ranged from −1.19 to 1.27.

### LCCIs of eyes with and without focal LC Defects

Table 2 compares LCCIs of eyes with and without focal LC defects. In all seven planes, LCCIs were significantly smaller in eyes with than without focal LC defects (all *P* values ≤ 0.001). The average LCCIs of eyes with and without focal LC defects were 9.75 ± 1.29 and 11.25 ± 1.39, respectively.

**Table 2:** LC curvature in eyes with and without focal LC defects.

LCCIs	All participants (n = 118)	Focal LC defect (n = 59)	Control glaucoma (n = 59)	*P* value
Plane 1	10.65 ± 2.03	9.74 ± 1.82	11.56 ± 1.83	**<0.001**
Plane 2	10.66 ± 2.10	10.09 ± 1.86	11.23 ± 2.19	**0.003**
Plane 3	10.39 ± 2.00	9.65 ± 1.89	11.13 ± 1.84	**<0.001**
Plane 4	9.66 ± 1.92	8.72 ± 1.62	10.59 ± 1.73	**<0.001**
Plane 5	10.37 ± 2.07	9.46 ± 1.80	11.29 ± 1.93	**<0.001**
Plane 6	10.98 ± 1.89	10.43 ± 1.67	11.54 ± 1.95	**0.001**
Plane 7	10.80 ± 1.81	10.17 ± 1.57	11.43 ± 1.82	**<0.001**
Average	10.50 ± 1.54	9.75 ± 1.29	11.25 ± 1.39	**<0.001**

LCCI = lamina cribrosa curvature index; LC = lamina cribrosa

Data are mean ± standard deviation values.

Bonferroni correction was applied to raw data for measurements in the seven planes. Values significant after Bonferroni correction (*P* < 0.0071; 0.05/7) are shown in bold.

### Distribution of parapapillary MvDs and focal LC defects

All MvDs were located within the PPA area. Of the 79 POAG eyes with an MvD, three and 73 had single MvDs in the superior and inferior hemispheres, respectively, and three eyes had large MvDs involving both the superior and inferior hemispheres. Interobserver agreement in the detection of MvD was excellent, with κ = 0.957. The ICC for measuring the area of MvD was 0.965.

**Figure 1:**
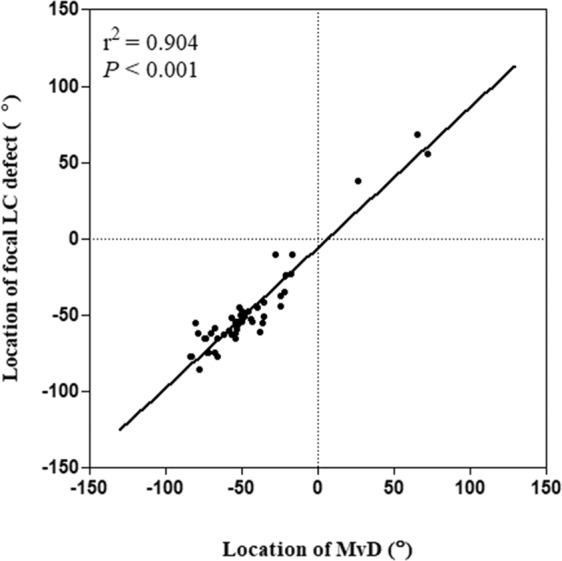
Scatterplot showing the topographic correlation between focal LC defects on SD OCT and microvascular dropout (MvD) on optical coherence tomography angiography in eyes with focal LC defect. Positive and negative locations of focal LC defects and MvDs indicate the locations that were superior and inferior to the foveal-disc axis, respectively.

Figure 1 shows the frequency distribution of MvD, which was consistent with the distribution of the focal LC defects.

### Factors associated with focal LC Defects

Univariate logistic regression analysis showed that the presence of an MvD (odds ratio [OR] = 0.174, *P* < 0.001) and smaller average LCCI (OR = 2.356, *P* < 0.001) were significantly associated with focal LC defects (Table 3). On multivariate analysis, the presence of an MvD (OR = 0.179, *P* = 0.001) and average LCCI (OR = 2.371, *P* < 0.001) remained statistically significant. Systolic BP (*P* = 0.065) and scan IOP (*P* = 0.074) were marginally significant on univariate analysis, but not on multivariate analysis (Table 3).

**Table 3:** Univariate and multivariate analysis factors influencing focal LC defects.

	Univariate			Multivariate		
Variables	Odds Ratio	95% CI	*P* value	Odds Ratio	95% CI	*P* value
Age, per 1-year older	1.000	0.966–1.036	0.979			
Gender, female	1.619	0.780–3.363	0.196			
Presence of DM	1.000	0.274–3.654	1.000			
Presence of HTN	0.617	0.279–1.362	0.232			
SBP, mmHg	0.971	0.942–1.002	0.065	0.988	0.951–1.026	0.522
DBP, mmHg	0.979	0.938–1.021	0.317			
MAP, mmHg	0.968	0.928–1.010	0.131			
MPP, mmHg	0.975	0.920–1.034	0.407			
Scan IOP, mmHg	0.853	0.716–1.015	0.074	0.875	0.706–1.085	0.224
**Presence of MvD**	5.758	2.332–14.215	**<0.001**	4.824	1.779–13.083	**0.002**
Area of MvD, mm^2^	18.873	2.387–149.250	**0.005**	0.687	0.151–17.557	0.687
Axial length, mm	1.023	0.743–1.409	0.877			
Central cornea thickness, μm	0.998	0.987–1.009	0.703			
Global RNFL thickness, μm	1.003	0.977–1.030	0.803			
Visual field MD, dB	1.003	0.947–1.062	0.921			
Visual field PSD, dB	1.044	0.958–1.137	0.330			
**Average LCCI**	0.425	0.298–0.606	**<0.001**	0.438	0.302–0.636	**<0.001**

DM = diabetes mellitus; HTN = hypertension; SBP = systolic blood pressure; DBP = diastolic blood pressure; MAP = mean arterial pressure; MPP = mean perfusion pressure; IOP = intraocular pressure; MvD = microvasculature dropout; RNFL = retinal nerve fiber layer; MD = mean deviation; PSD = pattern standard deviation.

Only variables with *P* < 0.1 on univariate analysis were included in the multivariate model.

Statistical significant factors are shown in boldface.

### Representative cases

**Figure 2:**
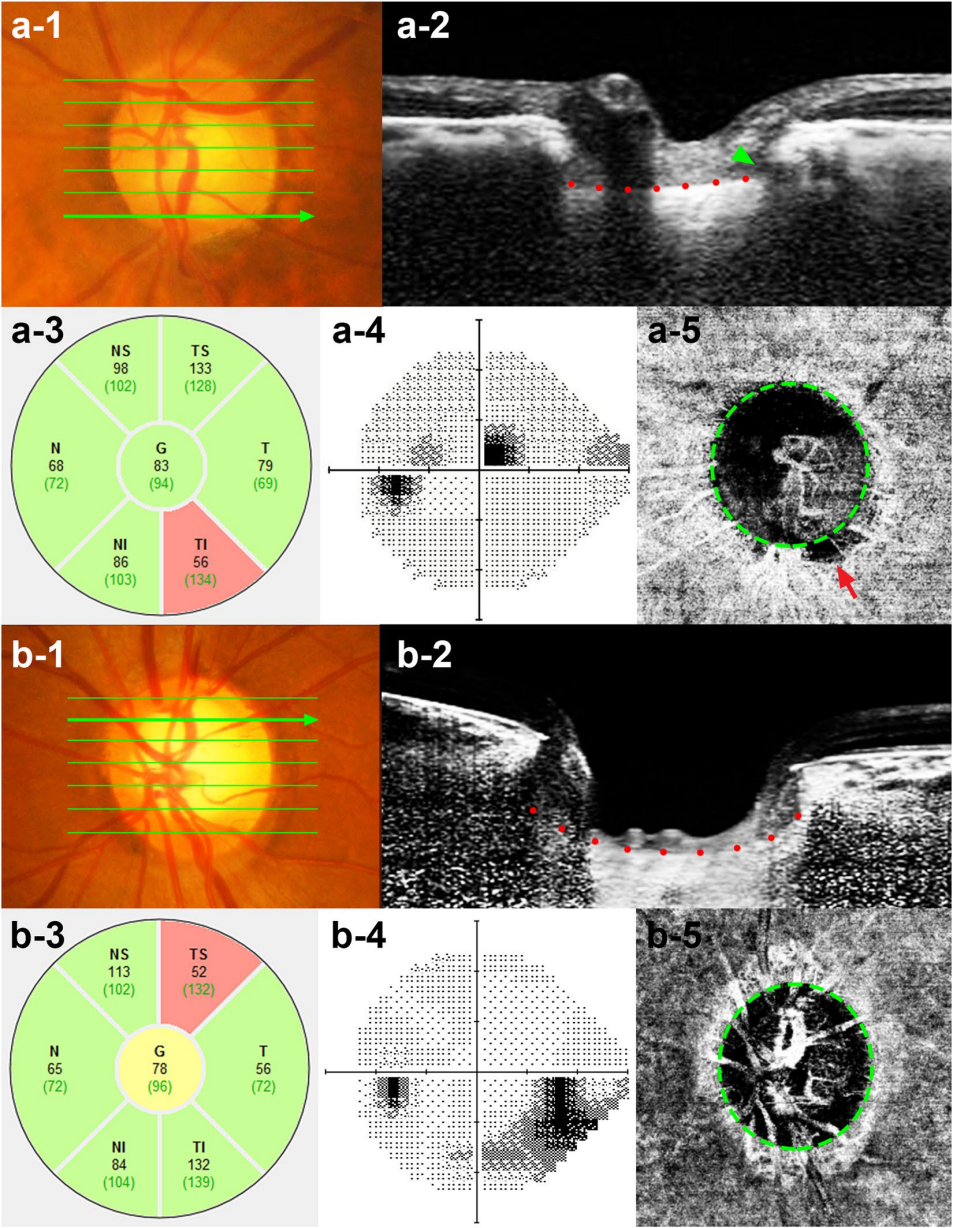
Representative eyes with (**a**) and without a (**b**) a focal LC defect. (a-1, b-1) Disc photographs of the left eye of a 75-year-old man (**a**), and a 50-year-old woman (**b**). (a-2, b-2) B-scan images obtained at the locations indicated by the *green arrows* in a-1and b-1, respectively. The focal LC defect is indicated by the *green arrow head* (b-2). Note that LCCI was smaller in the eye with (a-2, *red dots*) than without (b-2, *red dots*) a focal LC defect. However, retinal nerve fiber layer thickness (a-3, b-3) and visual field damage (a-4, b-4) did not differ between these two eyes. (a-5, b-5) *Green dashed lines* indicate the optic disc margin, and the *red arrow* indicates MvD (a-5). Note that the parapapillary MvD was located at the same sector as the focal LC defect.

Representative cases showing the differences in LCCI in eyes with and without focal LC defects are presented in Fig. 2. LC curvature was considerably smaller in the eye with (Fig. 2a) than without (Fig. 2b) focal LC defect.

## Discussion

This study demonstrated that the average LCCI and LCCIs measured in the seven horizontal B-scans were significantly smaller in glaucomatous eyes with than without focal LC defects when eyes were matched for age, axial length and glaucoma severity. To our knowledge, there has been no previous study in the literature investigating the relationship between generalized LC configuration and focal LC defects.

Previous studies have demonstrated posterior bowing of LC upon IOP elevation^4,5^ and reduction of LCCI after IOP lowering surgery^12^. These findings suggest IOP-induced stress is a primary driving force of generating and sustaining LC bowing. Therefore, it may be proposed that steeply curved LC is not simply an innate feature but determined by the accumulated change over time with age or due to glaucomatous remodeling, being influenced by the level of IOP^14^.

The LCCI has been used in many studies. We demonstrated excellent discriminating capability of LCCI between glaucomatous and healthy eyes^13^ and its better predictability for the rate of RNFL thinning than LC depth measurement^15^. More recently, correspondence between larger regional LCCI and location of RNFL defect has been demonstrated in POAG eyes with hemifield defect^14^. Taken together, these data indicate that LCCI is a valid indicator to evaluate the IOP-related, glaucomatous LC strain.

LC image was obtained using raster scanning. It is possible to measure LC curvature using vertical and radial scans, however, there are technical limitations for using those scan protocols. Because of the bowtie-shaped horizontal central ridge in the LC^16^, the LC would appear as “W-shape” in vertical scans. Therefore, LC curvature cannot be evaluated using a simple indicator like LCCI on vertical scans.

The average LCCI was found to be significantly smaller in eyes with than without focal LC defects. Several possibilities can be considered from this finding. First, given the association between IOP and LC curve^4,5,12^, the result suggests that IOP-related stress/strain is smaller in eyes with focal LC defects. Second, it is possible that posterior bowing of LC might have occurred in these eyes at the initial stage. The resulting posterior bowing may have induced severe stress in the laminar beams, particularly in the peripheral LC, which is known to more vulnerable to mechanical stress^17^, generating focal LC defects. After the development focal LC defects, the stress on given to the LC beams may have been canceled off and become flattened. This possibility was considered unlikely, however, because the LCCIs in the planes located at a distance from the focal LC defect (e.g., the superior optic disc plane in eyes with inferior focal LC defects) were also smaller. If IOP induced stress had been significant in those eyes, the LCCI would likely have been greater in the region away from the focal LC defect^14^. Third, the IOP stress may have been exerted in the transverse plane rather than the axial plane. As IOP increases, the LC may have become flattened due to scleral stretching of the eyeball, resulting intension pulling on both scleral openings^18^. Although this may be possible in some eyes, it is unlikely in the majority of eyes, because the LC typically bows posteriorly when IOP is elevated^4,5^ and becomes less curved after IOP lowering^12^.

Consistent with previous results^19^, the present study found the association between focal LC defects and MvD. OCTA defined MvD was found to coincide with perfusion defects on indocyanine green angiography^11^, suggesting that MvD indicates true circulatory impairment. Since the microvasculature both in the parapapillary choroid and the LC is supplied from the short posterior ciliary artery^20–23^, perfusion into the LC may be compromised. Taken together, these findings suggest that focal LC defects do not result primarily from IOP-related mechanical stress, but rather are associated with a degenerative process of the LC potentially initiated by compromised microcirculation to the LC. This finding supports the previous notion that circulatory impairment to the laminar region may be another reason for the structural change of the LC^24,25^. Primary deficiency in the blood supply to the laminar area could cause ultrastructural alterations of collagen and elastin that would serve to weaken the laminar beams and subsequently increase the vulnerability to deformation even at normal IOP^25,26^.

**Figure 3:**
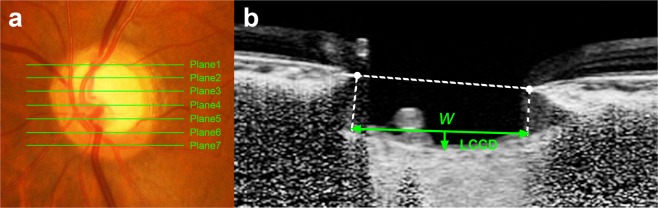
Measurement of the lamina cribrosa curvature index (LCCI). (**a**) Disc photograph showing seven horizontal planes (*green solid lines)* where the measurements were performed. (**b**) B-scan image obtained at plane 2 in (a). (**b**) The LCCI was measured by dividing the lamina cribrosa curve depth (LCCD) by the width of the anterior LC surface reference line (*W*) which connects the two points which meets the perpendicular lines from the Bruch’s membrane opening plane, then multiplying by 100.

The present study excluded myopic tilted disc eyes with gamma zone. This was due to two reasons. First, there is a technical problem in LC curvature measurement in tilted disc eyes. To measure the LC curvature, a vertical line from the Bruch’s membrane opening (BMO) was drawn from the reference line (Fig. 3)^13,27,28^. However, on the tilted disc, the vertical line cannot be drawn from the temporal BMO to the anterior LC surface because temporal BMO is located over the peripapillary sclera not on the LC. In addition, nasal anterior LC surfaces are often obscured by the shadow of thick nasal rim. Second, we consider that focal LC defect in myopic tilted eyes have unique pathogenic mechanism different from that in nontilted disc eyes. Tilted disc is often developed as an acquired feature due to scleral stretching along with axial elongation^29,30^. In this process, a tensile stress is applied between parapaillary sclera and the LC, resulting focal LC defect in the temporal LC^31–33^. Our interest was on the pathogenic mechanisms of focal LC defect which is not associated with such myopic tensile stress. For this purpose, including myopic tilted disc is not only inappropriate but also may induce biased interpretation on the role of vascular factor in the development of focal LC defect in nontilted disc eyes.

This study had several limitations. First, this was a cross sectional study. Therefore, the results of this study alone cannot establish that the LC defect is derived from the vascular mechanism. Longitudinal studies covering the entire period of focal LC development may be needed to confirm the relationships between the temporal sequences of structural LC and vascular changes in the parapapillary region and focal LC development. Second, all subjects included were Korean, indicating the need to study other ethnic groups. In addition, included patients have relatively mild-moderate glaucoma (mean VF mean deviation of −7.0 dB). Therefore, the results of this study may not be generalized to all patients. Third, myopic eyes were excluded, suggesting that these findings may be inapplicable to myopic eyes.

In conclusion, focal LC defects were not associated with steeply curved LC but with the presence of MvD in the adjacent parapapillary region. These findings support and extend the hypothesis that focal LC defects are primarily attributed to vascular mechanisms rather than by mechanical stress. Further prospective studies are needed to elucidate the precise mechanism of generation of focal LC defects.

## Methods

This investigation was based on an ongoing prospective study, the Investigating Glaucoma Progression Study (IGPS), being performed at the Seoul National University Bundang Hospital Glaucoma Clinic^34,35^. All eligible subjects provided written informed consent to participate. The study protocol was approved by the Seoul National University Bundang Hospital Institutional Review Board and followed the tenets of the Declaration of Helsinki.

### Study subjects

Each subject enrolled in the IGPS underwent comprehensive ophthalmic examinations including assessment of best-corrected visual acuity, Goldmann applanation tonometry, refraction, slit-lamp biomicroscopy, gonioscopy, dilated stereoscopic examination of the optic disc, stereo disc photography (EOS D60 digital camera, Canon, Utsunomiyashi, Tochigiken, Japan), and SD-OCT (Spectralis OCT, Heidelberg, Engineering, Heidelberg, Germany). The following measurements were performed in all subjects: corneal curvature (KR-1800, Topcon, Tokyo, Japan), central corneal thickness (CCT; Orbscan II, Bausch & Lomb Surgical, Rochester, NY, USA), and axial length (IOL Master version 5, Carl Zeiss Meditec, Dublin, CA, USA), as well as standard automated perimetry (Humphrey Field Analyzer II 750, 24–2 Swedish interactive threshold algorithm, Carl Zeiss Meditec) and OCTA (DRI OCT Triton; Topcon, Tokyo, Japan).

Subjects recruited in the present study were required to have primary open-angle glaucoma (POAG), a best-corrected visual acuity of at least 20/40, spherical refraction of −6.0 to +3.0 diopters (D), and cylinder correction of −3.0 to +3.0 D without a tilted appearance accompanied by gamma zone (defined as a tilt ratio of the longest to the shortest diameter of the optic disc >1.3)^36,37^ or torsion of the optic disc (defined as a torsion angle [the deviation of the long axis of the optic disc from the vertical meridian] > 15°)^37,38^, because it is highly likely that LC was distorted in these eyes. Subjects with a history of intraocular surgery except for cataract surgery, as well as subjects with retinal disease or neurologic disease that may affect visual field were excluded. Cases were also excluded in which a good-quality image (i.e., quality score >15) could not be obtained due to poor cooperation or the anterior border of the LC cannot be determined accurately on the obtained B-scans. If both eyes were eligible, one was selected randomly for inclusion. Untreated IOP was defined at the mean of five IOP measurements made on the same day (9AM to 5PM) or on different days before starting treatment to lower IOP. Scan IOP was defined as the IOP at the time of SD-OCT examination.

Systolic blood pressure (SBP) and diastolic blood pressure (DBP) were measured in the sitting position at the right upper arm with an automated oscillometric device at the time of SD-OCT examination^28^. Ocular systolic perfusion pressure (SPP) was defined as SBP – scan IOP and ocular diastolic perfusion pressure (DPP) as DBP– scan IOP. Mean arterial pressure (MAP) was calculated as DBP + 1/3 (SBP– DBP), and mean ocular perfusion pressure (MPP) as 2/3 (MAP) – scan IOP^28^.

POAG was defined as the presence of signs of glaucomatous optic nerve damage (i.e., diffuse or localized rim thinning, notching, or a disc hemorrhage) with an open iridocorneal angle on gonioscopy, corresponding visual field defect, and no identifiable secondary cause of glaucoma. A glaucomatous visual field defect was defined as a defect with one or more of the flowing criteria: (1) outside the normal limits on a glaucoma hemifield test; (2) three abnormal points with *P* less than 5% probability of being normal and one with *P* less than 1% by pattern deviation; or (3) a pattern standard deviation less than 5%, confirmed on two consecutive tests^39^. Visual field measurements were considered reliable when false-positive/negative results were less than 25% and fixation losses were less than 20%^39^.

### Enhanced depth imaging OCT of the optic nerve Head

The optic nerve and parapapillary area were imaged using the enhanced-depth-imaging technique of the Spectralis OCT system. The strengths and details of this technology for analyzing LC have been described previously^40^. Briefly, eyes were imaged through undilated pupils using a rectangle subtending 10° × 15° of the optic disc^1^. This rectangle was scanned with approximately 75 B-scan section images, which were separated by 30–34 μm, with the scan line distance determined automatically by the machine. Approximately 42 SD-OCT frames were averaged for each section. Using Spectralis OCT, the images were obtained only when the quality score was higher than 15. This protocol provided the best trade-off between image quality and patient cooperation^1^. The corneal curvature of each eye was entered into the Spectralis OCT system prior to scanning to avoid potential magnification errors.

### Quantification of posterior bowing of the LC

Following reconstruction of the 3D image, seven B-scan horizontal images that divided the optic disc diameter into eight equal parts vertically were selected for each eye. These seven B-scan lines were defined as planes 1 to 7, representing superior to inferior regions (Fig. 3). In this model, plane 4 corresponds to the mid-horizontal plane, and planes 2 and 6 correspond approximately to the superior and inferior mid-periphery, respectively^27^.

To quantify the posterior bowing of the LC, the LCCI was defined as the inflection of a curve representing a section of the LC, as described^13^. Briefly, the LC surface reference line was set in each B-scan by connecting the two points on the anterior LC surface that met the lines drawn from each Bruch’s membrane termination point perpendicular to the BMO reference line. The length of this reference line was defined as the width (*W*). The lamina cribrosa curve depth (LCCD) was defined as the maximum depth from this reference line to the anterior LC surface, and LCCI was calculated as (LCCD/*W*) ×100^27^.

The adaptive compensation was used to enhance the visibility of the peripheral LC prior to the measurement^41,42^. The LCCI was measured in each plane using a manual caliper tool provided by Amira software. LCCIs were measured by two experienced observers (SHL and EJL), who were masked to clinical information. The average LCCI for each eye was defined as the mean measurements at the seven planes.

### Assessment of the presence of focal LC defects

A focal LC defect was defined as an anterior laminar surface irregularity violating the normal smooth curvilinear U- or W-shaped contour^6^. To avoid false positives, defects had to be >100 μm in diameter and >30 μm in depth, and detectable in two neighboring horizontal B-scans^8^. The obtained SD-OCT images were independently reviewed by two observers (SHL and EJL) masked to all other clinical information, and the presence of focal LC defects determined. The B-scan locations were subsequently compared with the stereo disc photographs to confirm that any identified focal LC defects were not artifacts caused by vascular shadowing. The presence/absence of a focal LC defect was double checked using radial B-scans.

### Determination of the presence and area of parapapillary MvD

The optic nerve and parapapillary area were imaged using a commercially available OCTA device (Topcon), using the previously described protocol^43^. Briefly, scans were taken from 4.5 mm × 4.5 mm cubes, with each cube consisting of 320 clusters of four repeated B-scans centered on the optic disc.

The choroidal microvasculature in the parapapillary area was evaluated in the en face images of the parapapillary deep layer derived from an en face slab, extending from Bruch’s membrane to 390 µm below Bruch’s membrane, which was sufficient to include the full thickness of the choroid and inner sclera. MvD was defined as a focal sectoral capillary dropout without any visible microvascular network in the parapapillary area in en face OCTA images. An MvD was defined as a circumferential width of the area with capillary dropout greater than one half clock hour of the disc circumference^44^. MvDs were identified by two independent observers (SHL and EJL) who were blinded to the clinical information of the subjects. An MvD was considered identified only when both observers determined that it was present in the same sectoral location. Disagreements between these two observers were resolved by a third adjudicator (TWK). When the OCTA images were of poor quality, with blurring that hampered the delineation of MvD, the eye was excluded from the analysis. POAG eyes with multiple disjunct MvDs were excluded.

The area of each MvD was measured in square millimeters using the built-in manual drawing tool of the OCTA viewer software (V.1.21, IMAGEnet 6, Topcon; Fig. 2). MvD areas were measured by two masked observers (SHL and EJL) and averaged.

### Circumferential location of focal LC defects and MvD

**Figure 4:**
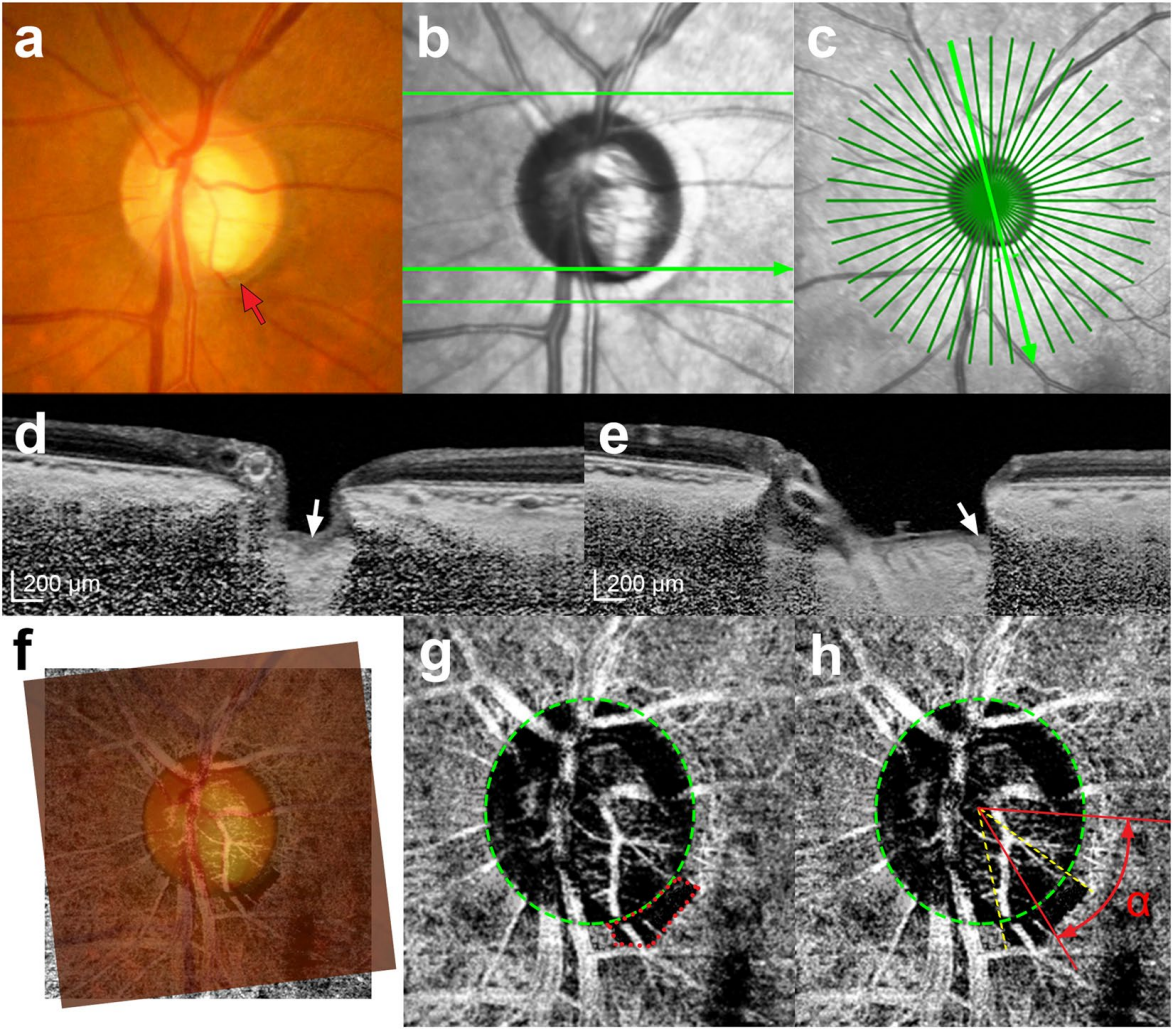
Evaluation of a focal lamina cribrosa (LC) defect and parapapillary microvasculature dropout (MvD). (**a**) Disc photography where the location of the focal LC defect was determined. (**b,c**) Infrared images indicating how the horizontal and radial scans were obtained. (**d**) Horizontal and (**e**) radial B-scan images obtained along the *green arrows* indicated in B and C, respectively. The *white arrows* indicate the location of the focal LC defect. (**f**) Combined image of a fundus photograph superimposed on the image obtained by optical coherence tomography angiography (**g**). (**g,h**) *Green dashed ellipses* indicating optic disc margins. MvD was defined as a focal sectoral capillary dropout with no visible microvascular network, and its area was measured by demarcation with the built-in manual drawing tool (g, *Red dotted line*). The location of the MvD was determined by measuring the angular distance of the midpoint of the MvD circumference relative to the foveal-disc center5 axis (h, α).

To determine the topographic correlation between MvD and a focal LC defect, the circumferential locations of both were measured. The circumferential location of the MvD was defined as the angular distance of the midpoint of the MvD relative to the foveal-disc axis, as described previously (Fig. 4h)^44^. To determine the foveal-disc center axis in SD-OCT and OCTA images, an infrared fundus image yielded at SD-OCT circumpapillary scanning and an en face OCTA image were superimposed and manually aligned on the red-free fundus photograph (Fig. 4f) separately, using commercial software (Photoshop CS6; Adobe Systems, Mountain View, CA, USA). The circumferential location of each focal LC defect was determined by measuring the angular distance from the fovea-disc center axis to the center of radial scans in which the focal defect was observed (Fig. 4). The locations of the MvD and focal LC defect were determined by two observers (SHL, and EJL), who were blinded to the participants’ clinical information, and averaged.

### Statistical analysis

To determine the inter-observer reproducibility of LCCI measurements, the Bland-Altman limits of agreement were used. The demographic and ocular characteristics of the two groups were compared using independent sample t-tests for continuous variables and chi-square tests for categorical variables. The topographic correlation of the location between focal LC defect and the MvD was evaluated by Pearson correlation analysis. Obtained *P* values from *t*-tests were subjected to Bonferroni’s correction, based on the number of comparisons. Factors influencing the focal LC defects were evaluated using logistic regression analysis. Statistical significance was considered when *P* values were less than 0.05. The Statistical Package for Social Sciences (version 22.0, SPSS, Chicago, IL, USA) was used for all statistical analyses.

## Supplementary information

Dataset 1.

## Data Availability

Data supporting the findings of the current study are available in Supplementary file.
